# Initial conservative treatment of osteochondral fracture of the patella following first-time patellar dislocation

**DOI:** 10.1186/s12891-020-03641-3

**Published:** 2020-09-17

**Authors:** Si Young Song, Tae-Soung Kim, Young-Jin Seo

**Affiliations:** Department of Orthopaedic Surgery, Hallym University Dongtan Sacred Heart Hospital, 7, Keunjaebong-gil, Hwaseong-si, Gyeonggi-do Republic of Korea

**Keywords:** Acute patellar dislocation, Medial patellar fracture, Osteochondral fracture, Redislocation

## Abstract

**Background:**

There has been no gold standard of the initial treatment strategy for acute patellar dislocation (APD) with osteochondral fracture (OCF). Hence the study aim is firstly, to review and compare clinical outcomes of patients who underwent conservative treatment for APD with or without OCF. Secondly, to characterize the location and size of fracture fragment.

**Methods:**

Sixty-nine consecutive patients who were retrospectively evaluated after first-time APD over a 2- year period were divided into two groups (group 1 (*n* = 24): APD with OCF and group 2 (*n* = 45): APD only). Magnetic resonance imaging (MRI) was used to assess patients with APD and OCF from the medial patella. All patients were treated with a supervised course of immobilization followed by progressive range of motion and strength exercise protocol. History of a recurrent dislocation, radiologic and functional scores were analyzed.

**Results:**

Redislocation rate was 31.2% in group 1 and 26.6% in group 2, showing no significant difference between the two groups (*p* = 0.690). Intergroup differences in terms of final Kujala and IKDC scores were not significant (*p* = 0.117 and *p* = 0.283, respectively). Fracture sites of the patella in group 1 were classified as follows: patellar medial margin (12), inferomedial facet (7), and inferomedial facet involving central ridge (5). In the subgroup of patient with OCF of the inferomedial facet of the patella, the fragments were found in the lateral gutter and did not cause pain or mechanical symptoms. Thus, loose body removal was not performed. However, all five patients with large OCF involving the central ridge of the patella failed non-operative treatment with recurrent dislocations, ultimately requiring fragment refixation and medial retinacular imbrication.

**Conclusions:**

First, APD patients with OCFs of medial margin or inferomedial facet showed similar redislocation rates and functional knee scores with those without OCFs after conservative treatment. Second, initial conservative treatment failed in some APD patients with large OCF, especially when OCFs were fractured from inferomedial facet involving central ridge. Surgery should be considered with this type.

## Background

Acute patellar dislocation (APD) is associated with a spectrum of soft tissue and osteochondral injuries. Based on multiple studies demonstrating similar functional outcomes after both conservative and operative procedures for treatment of APD, Conservative treatment has been traditionally the preferred option for APD without an osteochondral fracture (OCF) [1–8].

Redilocation is a significant concern in APD. Redislocation rate of APD ranges from 10 to 26% after surgical treatment and 13–52% after conservative treatment [3, 9–11]. Whereas, APD may be associated with OCF and the implications of this event on clinical outcomes are unclear. Studies on redislocation rate after various treatments of acute lateral patellar dislocation with OCF from the medial side of the patella are insufficient to draw solid conclusion regarding how it affects clinical outcome. Furthermore, the prevalence of APD with OCF fracture has not been well described yet. Although large OCF may be seen on plain radiographs, smaller fragments may go unrecognized.

There were various reports about treatment for APD with OCF, including conservative treatment, loose body removal and microfracturing, osteochondral fragment fixation, and medial patellofemoral ligament (MPFL) repair [12–16]. However, consensus for treatment has not been established yet due to limited research on surgical and non-surgical management for APD [3]. There has been no gold standard of the initial treatment strategy for APD with OCF [12–15, 17].

Thus, the first aim was to review and compare clinical outcomes of patients who underwent conservative treatment for APD with or without OCF. The second aim of this study was to characterize the location and size of OCF fragment. It was hypothesized that initial conservative treatment for APD with or without OCF would result in improved functional outcomes. APD patients with OCFs would show comparable redislocation rates and functional outcomes compared to those without OCFs after conservative treatment.

## Methods

This was a retrospective study of 69 consecutive patients with mean age of 19.6 years (range, 15 to 41 years) who were followed up over a 2-year period after first-time APD. The study was approved by IRB (institutional review board)/EC (Ethics committee) of our hospital (approval number: HDT 2019–06-016). Patient inclusion criteria were: (1) a diagnosis of first time APD based on results of magnetic resonance imaging (MRI, MagnetomSkyra, SIEMENS, PA, USA) and SOMATOM Sensation high-resolution computed tomography (CT) scanner (Siemens MagnetomVerio, SIEMENS, PA, USA) that revealed evidence of APD such as medial retinacular injury, hemarthrosis, medial patellar and lateral femoral bone contusion, [18] or patellar OCF (MPFL bony avulsion or medial patellar OCF), (2) presentation less than 4 weeks from injury, (3) patients who received conservative treatment initially, (4) no evidence of patellofemoral osteoarthritis to rule out degenerative loose body formation, and (5) follow-up of at least 2 years after initial presentation. Exclusion criteria were: (1) APD patients without MRI evaluation to exclude potential existence of OCF which was invisible on simple X- ray, (2) patients who were suspected to have only subluxation history without typical MR findings of lateral patellar dislocation [18]. (3) APD patients with femoral side osteochondral avulsion injury, or (4) medial patellar dislocation.

Radiographic examination included weight bearing antero-posterior with full extension, Rosenberg view, and merchant view to exclude patellofemoral osteoarthritis which might cause potential selection bias. Patellar height was measured on lateral radiograph using published method described by Insall and Salvati [19]. Lower limb alignment was assessed using standing scanogram. Trochlear sulcus angle and tibial tuberosity trochlear groove (TTTG) distance were evaluated on axial images of MRI. TTTG distance was measured on superimposed axial sections as described by Dejour [20, 21].

Simple x-ray, CT, and MRI were carefully evaluated to inspect existence of medial patellar OCF. If this was the case, original location of the fracture fragment on the patella was then evaluated. Cases with OCFs were allocated into group 1 (24 cases). Others were allocated into group 2 (45 cases). In this study, the medial patellar OCF was defined as bony avulsion from the medial margin of the patella, OCF from inferomedial facet or OCF from inferomedial facet involving central ridge, where there was discontinuity between the fracture fragment and the medial side of the patella that was confirmed by MRI and CT. CT was also used to characterize original location of the fracture fragment.

All patients were recommended to perform protective weight bearing for 4 weeks with knee brace and crutches. Meanwhile, continuous passive motion (CPM) and passive range of motion with heel gliding were allowed as tolerated. No strengthening exercise was started until the 4 weeks point after initial injury. After that, closed kinetic chain exercise were started and progressed to open chain kinetic excise and full strengthening exercise. Return to previous sports activities were allowed in 6 months after initial injury. Whereas, some of the patients with large OCFs (five patients with OCFs of the inferomedial facet involving central ridge) had finally failed of conservative treatment due to recurrent patellar dislocation episodes during conservative treatment protocol. These five cases were not included in the final outcome analysis because surgery was performed during follow-up period.

History of a recurrent dislocation after initial presentation and knee functional scores at the last follow-up were retrieved from medical record or telephone survey. Redislocation was defined as a recurrent lateral patellar dislocation that required a further visit to the hospital [22]. Functional outcomes of each group were assessed using the Kujala score and International Knee Documentation Committee Subjective Knee Form (IKDC SKF) questionnaire at initial presentation and final follow-up [23, 24].

### Statistical analysis

Paired T-test was used to compare functional knee scores between initial and final follow-up data. Variables regarding intergroup comparison were analyzed using Chi-squared test and Student T-test. The level of significance was set at *p <* 0.05. All analyses were performed using SPSS for Windows (version 21.0, SPSS Inc., Chicago, IL, USA). A priori power analysis was performed with G*Power (v3.1.2) to determine the number of patients needed for tests of two independent proportions (redislocation rate). Based on previous literatures, [9, 10, 14, 15] the value of two proportions were defined as 0.55 for group 1 and 0.20 for group 2 and allocation ratio was defined as 2.2. A sample size analysis with a power of 80% and an alpha of 0.05 showed that 17 subjects for group 1 and 36 for group 2 were required. Hence, 19 patients for group 1 and 45 patients were identified as enough to detect significance between the two groups.

## Results

### Basic demographics

During the study period from October 2012 to December 2018, medical records of 87 patients with first time acute patellar dislocation were identified. Of those, 18 patients were excluded for the following reasons: one patient with medial dislocation, two patients with only subjective subluxation episode, seven patients with femoral side osteochondral injury and eight patients whose data were unavailable at the minimum two-year follow-up examinations. Thus, 69 patients including 42 males and 27 females met inclusion criteria.

**Fig. 1:**
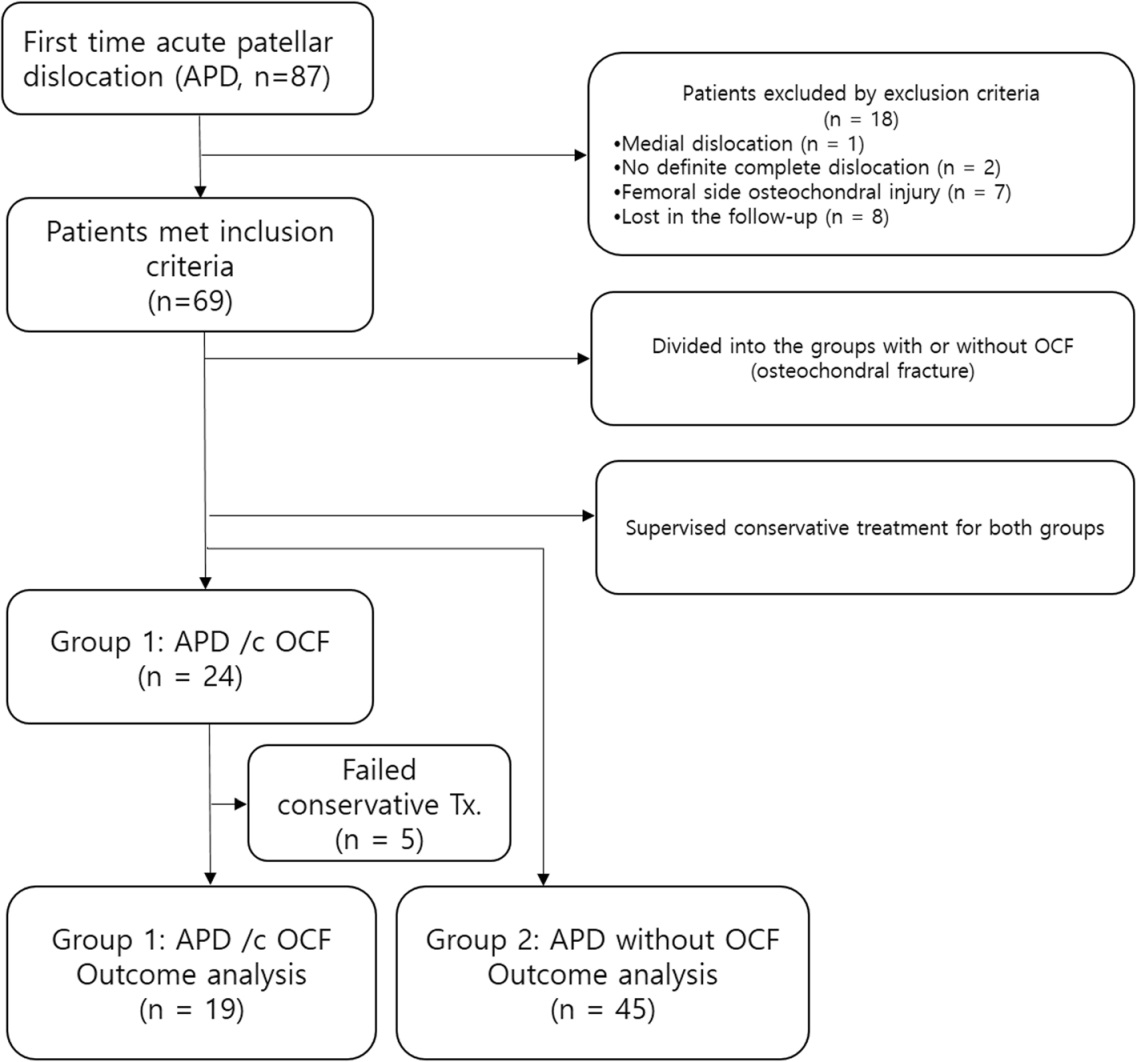
Flow chart of patients studied

Their mean age was 19.6 years (range, 15 to 41 years; Group 1: 19.1 ± 3.9 years; Group 2: 19.9 ± 5.4 years) with no significant difference between the two groups (*p* = 0.486). All patients were identified during the study period with a minimum follow-up period of 24 months and a maximum follow-up period of 48 months (mean 32.3 ± 7.5 months). Twenty-four patients (group 1) had APD with OCF, whereas the other 45 patients (group 2) had APD only. As described above, five patients of group 1 who had large OCF involving the central ridge of the patella were not included in the outcome analysis because they failed non-operative treatment due to recurrent dislocations despite supervised conservative treatment protocol, ultimately requiring surgery (Fig. 1).

### Radiologic demographics

The mean Insall-Salvati ratio was 1.2 ± 0.2 for group 1 and 1.3 ± 0.2 for group 2, showing no significant difference between the two groups (*p* = 0.082). The mean TTTG distance was 16 ± 5.7 mm for group 1 and 15 ± 4.2 mm for group 2, showing no significant difference between the two groups (*p* = 0.125). Trochlea sulcus angle was measured to be 152 ± 12.8 ° for group 1 and 151 ± 13.5 ° for group 2, showing no significant between-group difference (*p* = 0.346). The mean hip-knee–ankle angle as measured on a standing scanogram was valgus 3 ± 4.5 ° for group 1 and varus 1 ± 6.7 ° for group 2 (*p* = 0.035).

### Osteochondral fracture

**Fig. 2:**
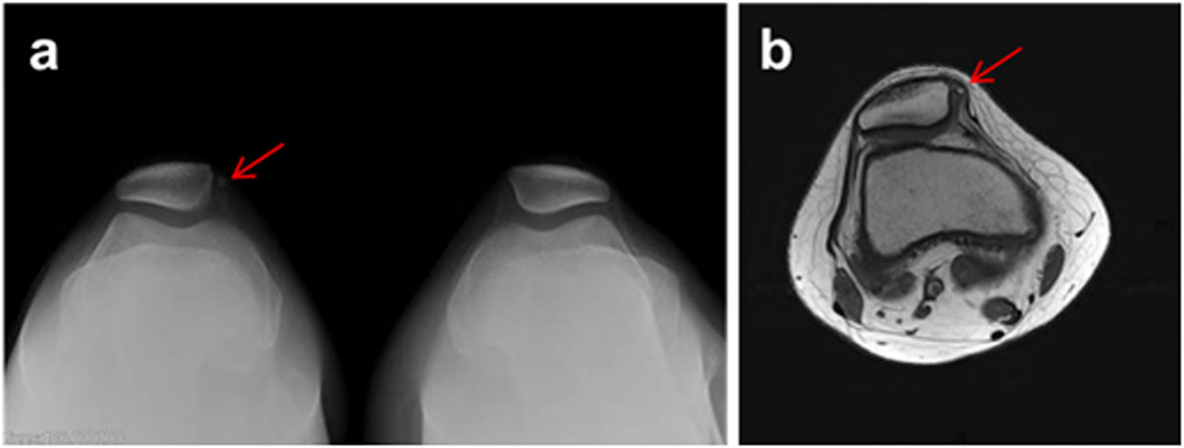
**a** A 15-year-old male sustained after an APD with bony avulsion on the medial patellar margin. A, Merchant view showing a bony avulsion (arrow) beside right patella medial margin. **b** Axial T2-weighted image showing a fracture fragment on medial aspect of the patella (arrow)

**Fig. 3:**
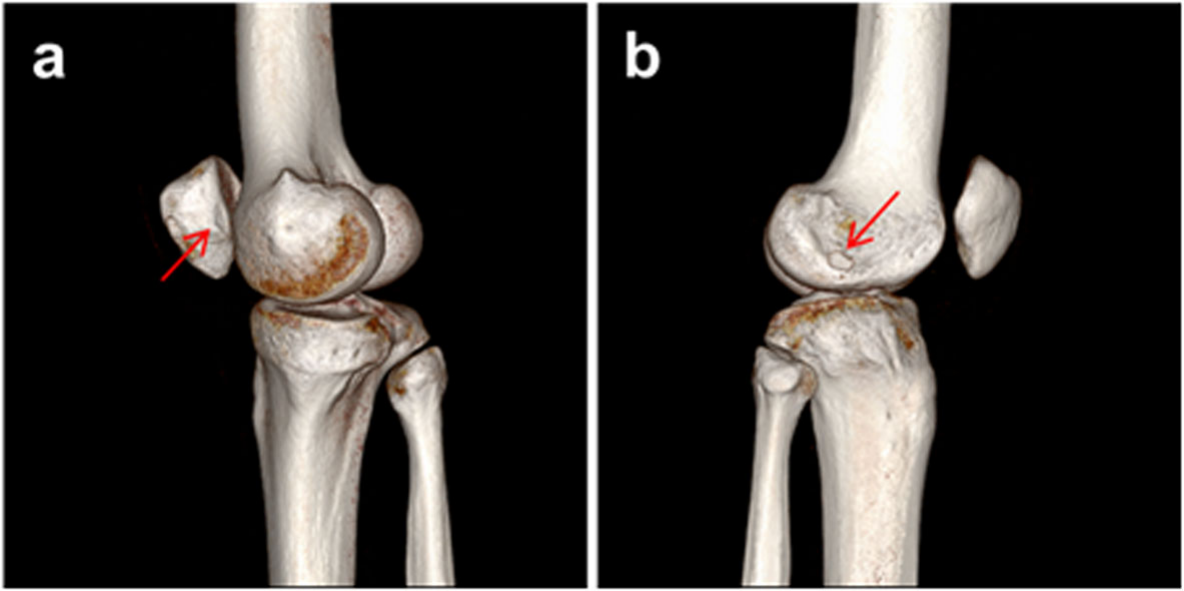
**a** A 25-year-old male with OCF from the inferomedial facet which may be a consequence of impact between lateral femoral condyle (LFC) and medial patellar facet during relocation of the dislocated patella. Osteochondral defect on inferomedial facet of the patella (arrow). **b** Osteochondral fragment on the lateral surface of LFC (arrow)

**Fig. 4:**
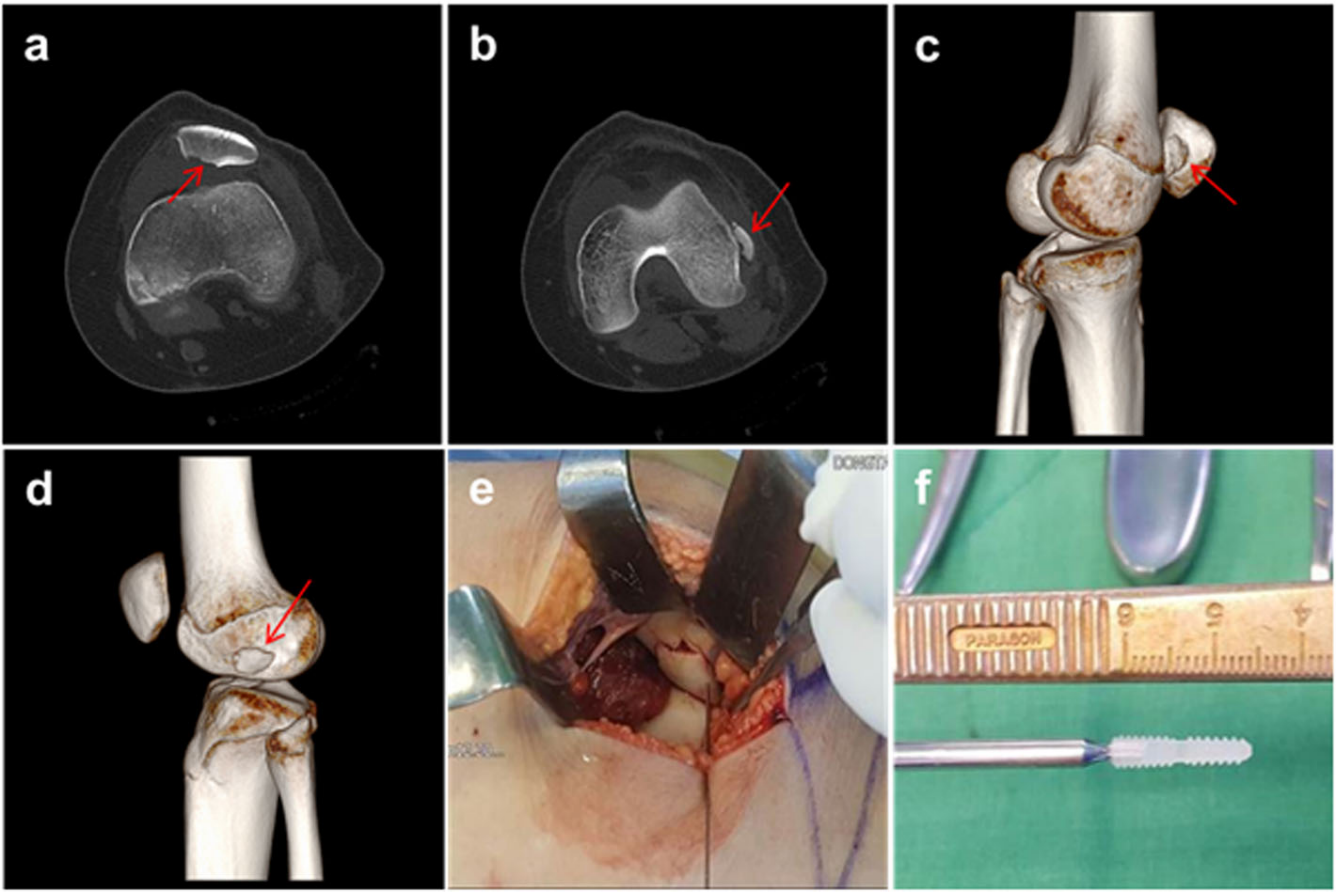
A 19-year-old female with OCF from the inferomedial facet involving central ridge. **a** A CT axial image showing osteochondral defect on the patellar inferomedial facet involving central ridge (arrow). **b** Osteochondral fragment next to LFC (arrow). **c** A 3D-CT reconstruction image showing osteochondral defect on the patella inferomedial facet involving central ridge (arrow). **d** Osteochondral fragment next to LFC (arrow). **e**, **f** Arthroscopic retrieving the fragment and open refixation of the fragment with a biodegradable headless screw

In terms of medial patellar OCF fragment, it was classified based on the location of fracture site, patellar medial margin, inferomedial facet, and inferomedial facet involving central ridge [25]. Of 24 patients with medial patellar OCF, 12 had bony avulsion on the medial margin of the patella, suggesting MPFL avulsion fracture from the patellar attachment (Fig. 2). Another twelve OCFs were originally located on the patellar inferomedial facet (Fig. 3), five of which were inferomedial facet involving central ridge (Fig. 4). The mean size of the OCF fragments from the inferomedial facet was 16 (major axis) × 12 mm (minor axis). Wheareas, the mean size of OCF fragments from inferomedial facet involving central ridge was 28 mm × 20 mm.

### Clinical & functional outcomes

**Fig. 5:**
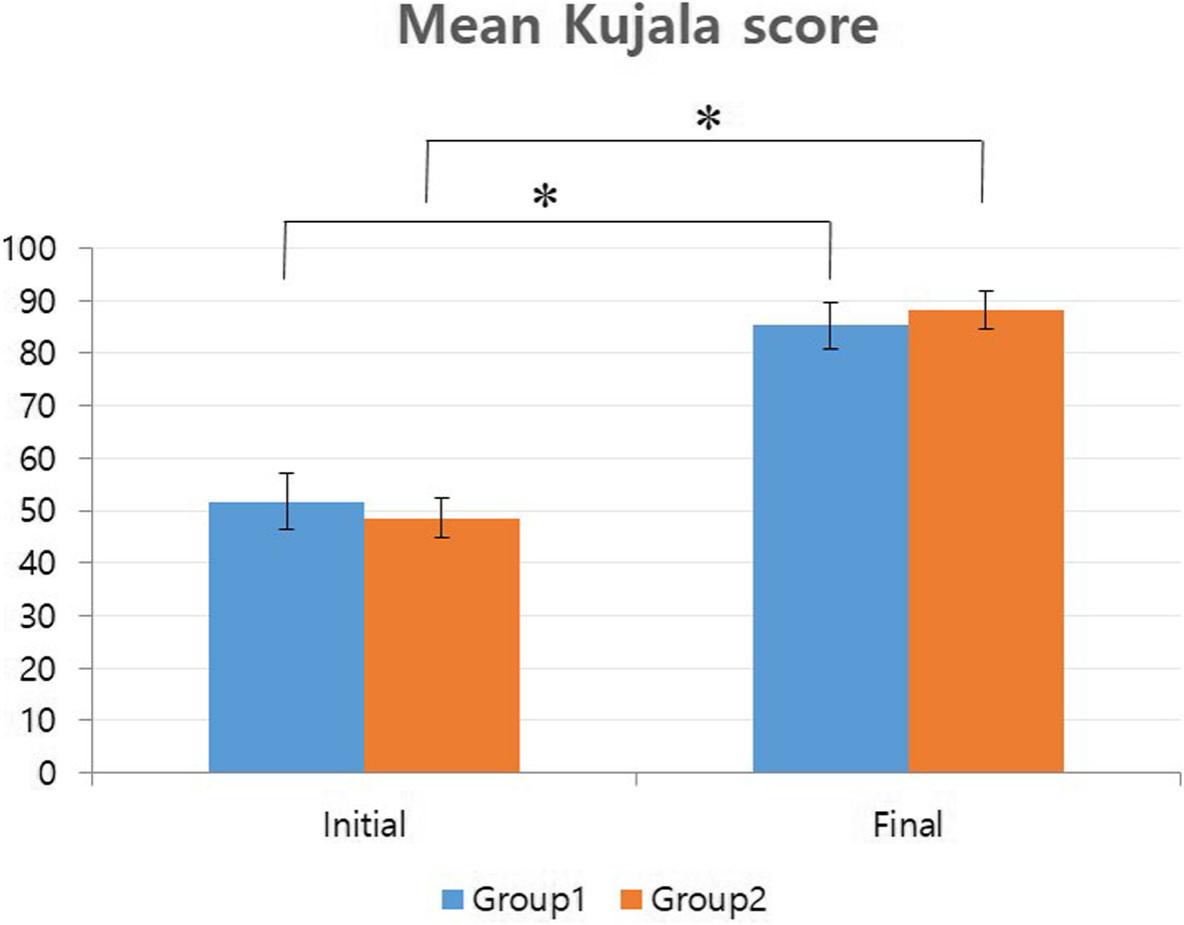
Mean Kujala scores between group 1 and group 2. * Denotes statistical significancy. Intergroup difference in terms of final IKDC SKF was not significant

**Fig. 6:**
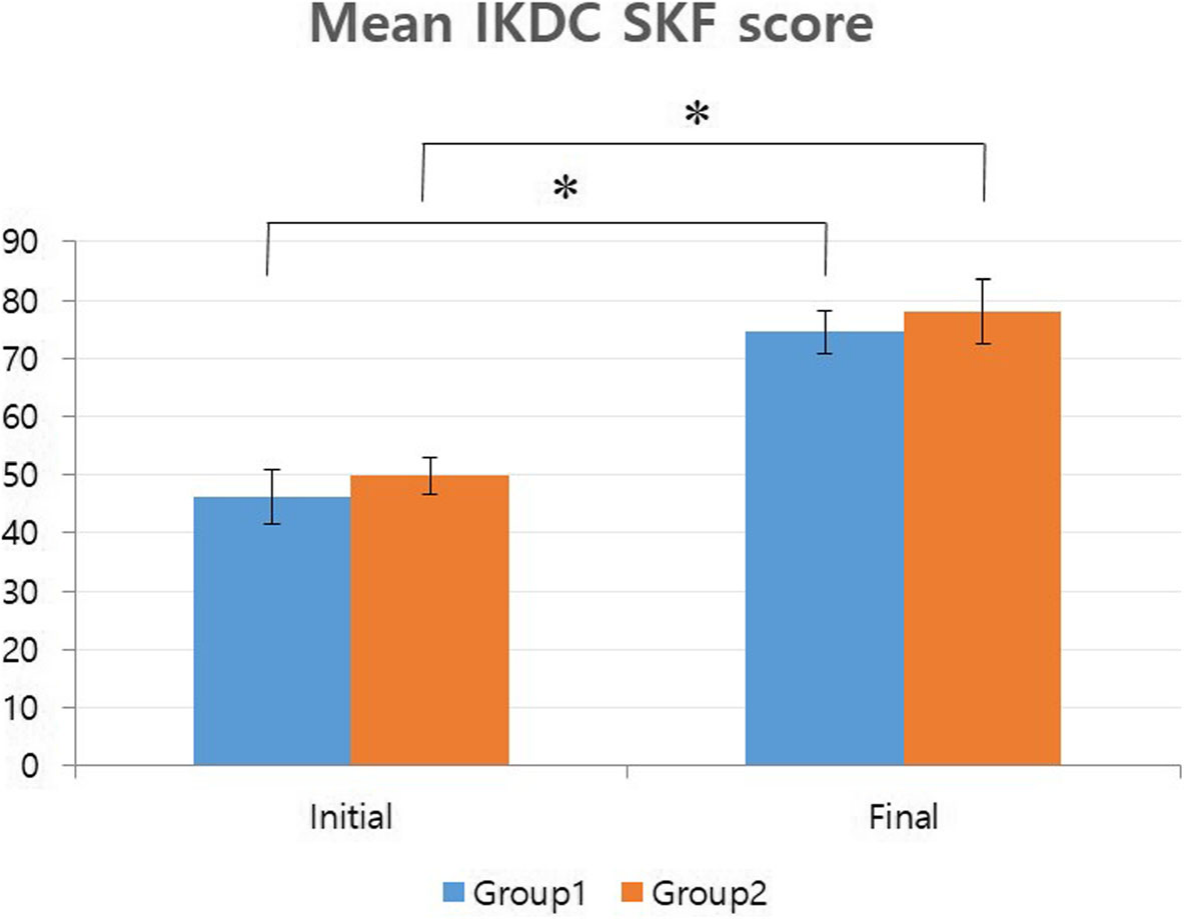
Initial and final IKDC scores between group 1 and group 2. * Denotes statistical significancy. Intergroup difference in terms of final IKDC SKF was not significant

In terms of redislocation rate, it was 31.2% for group 1 and 26.6% for group 2, showing no significant difference between the two groups (*p* = 0.690). When the five patients with the large OCFs involving the central ridge were included in the outcome analysis of redislocation rate (all of the five patients were regarded as redislocation cases of group 1), the intergroup difference of redislocation rate was also insignificant (*p* = 0.108). The initial mean Kujala score in group 1 was 51.7 ± 10.5. It was significantly increased to 85.3 ± 8.8 at the final follow-up (*p* < 0.001). Mean Kujala score for group 2 was also increased significantly from a mean of 48.6 ± 7.5 at initial presentation to 88.3 ± 7.4 at the final follow-up (*p* < 0.001). Statistically significant differences in the final Kujala scores between the two groups were not detected (*p* = 0.117) (Fig. 5). The mean IKDC SKF for group 1 significantly improved from initial 46.3 ± 9.3 to final 74.5 ± 7.3 (*p* < 0.001). Group 2 also exhibited a significantly improved score at final follow-up compared to the initial score (from mean of 49.8 ± 6.5 to 78.0 ± 11.2, (*p* < 0.001)) (Fig. 6). Intergroup difference in terms of final IKDC SKF was not significant (*p* = 0.283).

## Discussion

The present study had the following important findings. First, APD patients with OCFs of medial margin or inferomedial facet showed similar redislocation rates and functional knee scores with those without OCF after supervised conservative treatment. In addition, final functional knee scores significantly improved from the base line in both groups. And the final scores were not significantly different between the two groups. Our hypotheses were partially confirmed. Second, initial conservative treatment failed in some APD patients with large OCF, especially when OCFs were fractured from inferomedial facet involving central ridge. Initial surgery should be considered for patients with OCF of this type. To the best of our knowledge, this is the first known series to compare clinical outcomes after initial conservative treatment following APDs with or without medial patellar fracture.

The patella is at risk for fracture at both the time of dislocation (OCF from the medial margin) and during relocation (OCF from the inferomedial facet). This may in part account for the higher incidence of patellar osteochondral injury. Osteochondral injury to the patella is a relatively common occurrence following lateral patellar dislocation of the knee [13]. In this study, OCF from the patellar medial margin was defined as bony avulsion on the medial margin of the patella. And the fracture was considered to occur by the pull of MPFL from the patellar attachment during lateral dislocation of the patella. Wheareas, as the dislocated patella is reduced from a lateral to a medial direction by the pull of vastus medialis, the medial facet of the patella can impact the lateral femoral condyle which may result in fracture, leaving the fracture fragment in the lateral gutter [16].

In the past, articular cartilage injuries were considered to occur less frequently. In the study reported by Rorabeck et al., OCFs occurred in 18 patients (5% of their APD patients) aged 12 to 18 years [26]. Various other authors have reported a 10 to 30% incidence of intra-articular osteochondral injuries seen on radiographs after APD [17, 26–30]. However, recent studies have shown articular cartilage injuries in 40 to 76% of APD patients arthroscopically, at surgery, or on MRI [17, 31–33]. In the present study, APD with OCF occurred in 35% of total subjects, similar to recent studies.

Silanpaa et al. have reported characteristics of MPFL avulsion injury at patellar attachment. They found three types of patellar MPFL injuries, ligamentous disruption, medial margin bony avulsion, and bony avulsion involving articular cartilage margin [14]. Nomura et al. have reported patterns of articular cartilage injury of patella based on arthroscopic finding. They found that 28 (72%) of 39 APD knees showed cartilage defects caused by OCF. The main site of cartilage defect was found to be the medial facet including odd facet. The inferior half of the medial facet was the most common site [25]. In the present study, locations of defects were patellar medial margin, patellar inferomedial facet, and inferomedial facet involving central ridge.

Traditionally, conservative treatment strategy for APD has been suggested because surgery is not superior to nonsurgical treatment based on systematic review and randomized studies [3, 34–36]. whereas, consensus for treating APD with OCF has not been established yet because of limited research comparing surgical and non-surgical initial management for people having dislocated patella with OCF [3].

Some surgeons have reported that patellar MPFL avulsion in primary traumatic patellar dislocation does not benefit from acute surgical repair compared to nonsurgical treatment because of statistically non-different redislocation rate and functional knee scores [14]. Previous retrospective comparative study between fixation and non-fixation for OCF after APD with OCF reported that similar redislocation rate between two groups and better functional knee score in non-fixation group [13]. Similar outcomes were reported by Seeley et al. In their retrospective study with 10-year period follow-up, chondral defect involved in the medial patella was found in 35 patients of a total of 122 patients who had sustained APD. Twenty (57%) of 35 patients with patellar chondral defects underwent surgical intervention such as isolated loose body removal or combined medial structure repair. They reported no statistical difference between the medial structure repair group and non-repair group in terms of IKDC scores. They also concluded that mode of treatment did not significantly affect rate of recurrence [16].

Based on above-mentioned studies reporting similar clinical outcomes and redislocation rate after APD with OCF between surgical and non-surgical treatment, these findings motivated us to hypothesize that conservative treatment could be reasonable treatment option for treating APD with OCF. Our results showing that redislocation rate in APD patients with OCFs from medial margin and inferomedial facet was similar to that in APD patients without OCF following conservative treatment partly concurred with a previous report [13, 16].

However, treatment algorithm should be individualized based on various scenarios of osteochondral injury, because some surgeons have reported a better stability after surgical reinsertion of MPFL than that after non-operative treatment in case of high failure rate such as significant patellar MPFL osteochondral fracture [14, 34–37]. Failure of conservative treatment in case of OCF involving inferomedial facet with central ridge was shown in this study. Thus, initial surgery should be considered for such cases. The mean size of OCFs in these failed cases was 28 × 20 mm. For these cases, we performed arthroscopic retrieving for the fragment and open refixation of the fragment with biodegradable headless screws on the original fracture site of the patella. And medial imbrication was done (Fig. 4). Mean Kujala score and IKDC SKF in these five cases improved from 43.2 ± 5.7 and 45.5 ± 6.4 to 87.3 ± 4.4 and 75.4 ± 8.2, respectively. There was no recurrence dislocation during minimum follow-up of 2 years.

In the case of OCF from inferomedial facet of patella, OCF is located in the lateral gutter (Fig. 3). This fragment, as mentioned earlier, is the result of collision with the lateral aspect of the lateral condyle. Although this OCF was considerably displaced from the patella, most of it was attached to the lateral surface of the lateral femoral condyle, which was not the weight bearing surface. None of the OCFs examined in this study caused mechanical symptoms due to joint incarceration. And the OCF fragments did not migrate into tibiofemoral and patellofemoral articulation during follow-up. Thus, loose body removal procedure was not needed.

This study has some limitations. First, Although the sample size was large enough to show a statistically significant difference between the groups, the number of patients was small. This small sample size was inevitable because of delicate inclusion criteria. However, despite the small numbers, our results do suggest that patients with OCF including the central ridge of the patella are at higher risk of dislocation with non-operative treatment. We are hopeful that future prospective studies could further assess this relationship and assist surgeons in making treatment decisions for first time APD. Second, the follow-up period was relatively short. Thus, results could not reflect mid- or long- term outcomes. As time passes, there would be a potentially greater risk of further instability that would require surgical treatment. Third, there might be a selection. Bias because of its retrospective nature. However, this study was meaningful in that it performed intergroup comparison in which homogenous intervention was performed. In addition, all subjects underwent conservative treatment for initial treatment. Lastly, there was demographic difference in limb alignment between groups 1 and 2, but it is unclear what effect this would have on successful nonoperative management.

## Conclusion

In the case of APD with OCF fractured from the medial margin and inferomedial facet, similar functional outcomes and redislocation rates after conservative treatment were shown compared with the APD only group. Whereas, initial conservative treatment failed in some APD patients with large OCF, especially when OCFs were fractured from inferomedial facet involving central ridge. Surgery should be considered with this type.

## Data Availability

The datasets used and analyzed during the current study are available from the corresponding authors on reasonable request.

## Abbreviations

APD: Acute patellar dislocation
OCF: Osteochondral fracture
MPFL: Medial patellofemoral ligament
MRI: Magnetic resonance imaging
CT: Computed tomography
TTTG: Tibial tuberosity trochlear groove
CPM: Continuous passive motion
IKDC SKF: International knee documentation committee subjective knee form
